# Fusion of [^18^F]FDG PET with Fluorescence Diffuse Optical Tomography to Improve Validation of Probes and Tumor Imaging

**DOI:** 10.1007/s11307-012-0581-z

**Published:** 2012-08-28

**Authors:** Anikitos Garofalakis, Albertine Dubois, Benoît Thézé, Bertrand Czarny, Bertrand Tavitian, Frédéric Ducongé

**Affiliations:** 1 CEA, Institut d’Imagerie Biomédicale, Service Hospitalier Frédéric Joliot, Orsay Cedex, France; 2 INSERM U1023, Laboratoire d’Imagerie Moléculaire Expérimentale, Orsay Cedex, France; 3 Université Paris Sud, Orsay Cedex, France; 4 CEA, Institut de Biologie et de Technologies de Saclay, Service d’Ingénierie Moléculaire de Protéines, Gif/Yvette Cedex, France

**Keywords:** Fluorescence molecular tomography, Fluorescence diffuse optical tomography, Positron-emission tomography, Small-animal multimodal imaging, Cancer

## Abstract

**Purpose:**

Given the progress of fluorescence diffuse optical tomography (fDOT) technology, here, we study the additional benefits provided by multimodal PET/fDOT imaging by comparing the biodistribution of 2-deoxy-2-[^18^F]fluoro-d-glucose ([^18^F]FDG) in tumors with three fluorescent probes: a glucose analog, a protease activatable optical probe, and a ligand of αvβ3 integrin.

**Procedures:**

Sequential fDOT/PET/computed tomography (CT) imaging of mice was performed with a custom multimodal mouse support that allows the subject to be transferred between the fDOT and the PET/CT scanners. Experiments were performed in xenografted tumor models derived from the human breast cancer line MDA-MB 231 and compared to *ex vivo* analysis.

**Results:**

The three-dimensional signals showed that the fluorescent glucose analog is not colocalized with [^18^F]FDG, raising questions about its use as a surrogate probe of the PET tracer. Fusion of [^18^F]FDG with the other fluorescent probes showed evidence of high variability both for the protease activity and the αvβ3 integrin expression during tumor growth.

**Conclusion:**

The added value of hybrid PET/fDOT over the two modalities was demonstrated for cross-validation of probes and for better characterization of tumor models.

## Introduction

In oncology, 2-deoxy-2-[^18^F]fluoro-d-glucose ([^18^F]FDG) is largely used to monitor the glucose metabolism of tumors by positron-emission tomography (PET) imaging. Indeed, many tumors exhibit a higher uptake of [^18^F]FDG compared to most normal organs or tissues because cancer cells usually overexpress glucose transporter (GLUT) proteins and have increased levels of active hexokinase [1]. Hence, [^18^F]FDG PET imaging is now routinely used in clinics for the evaluation of several neoplasms, both before and after therapy, helping to stage tumors and monitor tumor response [2]. Similarly, [^18^F]FDG PET imaging has been increasingly used in preclinical research for both basic research and drug development [3, 4]. However, it is well known that cancer is a multistep and multiparametric disease [5, 6]. Therefore, there is an increasing demand to simultaneously study glucose metabolism in combination with other factors, such as hypoxia, tissue remodeling, or angiogenesis. Such multimodal imaging can be particularly useful to study the effect of a therapy or the function of a specific oncogene to the development of tumors in small animal models.

To address this issue, several PET tracers have been developed to highlight different physiological processes of tumors [2]. However, the coregistration of [^18^F]FDG with another PET tracer is limited since tracers have to be injected sequentially, waiting the decay of the previously injected isotope without moving the animal. Another possibility is to fuse [^18^F]FDG PET with information originating from another imaging modality. So far, PET imaging has been fused with different imaging modalities, mostly X-ray computed tomography [7] (CT) and, more recently, magnetic resonance imaging [8] (MRI). However, although CT and MRI display high-spatial resolution imaging, they lack contrast agents to perform functional imaging and are mostly used to measure tumor's morphologic features (*i*.*e*., size, shape, or blood vessel density).

Recently, optical techniques have been increasingly used for small animal imaging. However, the fusion of PET with these new modalities of imaging has not been very developed so far. This could be explained by the fact that most optical imaging systems are currently used to perform planar imaging as opposed to three-dimensional (3D) imaging. The signal detected by such two-dimensional (2D) systems is strongly affected by scattering of light in tissue and can only provide semiquantitative measurements from 2D images. To address this issue, fluorescence diffuse optical tomography (fDOT) technique, also known as fluorescence molecular tomography, has been developed since the 1990s by several groups [9–13]. This technology employs instruments that operate in a transillumination excitation mode and uses sophisticated reconstruction algorithms [10] for reconstructing a 3D fluorescence signal with similar resolution as PET. Although, fDOT is still only able to perform imaging inside tissue a few centimeters in depth, it is perfectly adapted to *in vivo* imaging in mice. Therefore, fDOT systems dedicated to small animals have considerably evolved in less than 10 years, from systems in which the animal was immersed inside an index matching fluid [13] to systems where the animal was not immersed, but still compressed between two transparent plates [14] and, finally, to contact-free systems [15]. Our group developed a simple method to fuse small animal PET and CT imaging with fDOT. This method uses a mouse support with a transparent plexiglas plate that can be moved between all the three different imaging modalities and a dedicated software that allows coregistration of independently acquired images [16]. In a previous study, we calibrated a contact-free fDOT apparatus by using this method for correlating its measures with quantitative values obtained by PET, as a gold standard. Our results clearly demonstrated the accuracy of fDOT to quantify the biodistribution of probes inside mice, in a manner comparable to PET for concentrations ranging from 3 nM to 1 μM [16]. A similar correlation between PET and fDOT imaging was also demonstrated by Nahrendorf *et al*. [17], using another fDOT system where the animal is compressed between transparent plates.

In addition of validating fDOT apparatus, these results prompted us to evaluate the additional benefits of fDOT/PET imaging in other applications. Here, we use fDOT/PET to complement [^18^F]FDG PET imaging of a tumor xenograft with three different near-infrared (NIR) fluorescent probes: (1) a fluorescent labeled glucose analog, (2) a protease activatable probe, and (3) a fluorescent probe targeting the αvβ3 integrin. These studies were conducted to demonstrate the additional benefits of combined fDOT and PET imaging for cross-validating probes and highlighting different tumor processes in parallel inside the same subject.

## Materials and Methods

### Cell Culture

Human breast cancer MDA-MB-231 cells were grown in Dulbecco's modified Eagle’s medium supplemented with 10 % fetal calf serum, l-glutamine (2 mM), penicillin (100 U/ml), and streptomycin (100 μg/ml) at 37 °C in a 5 % CO_2_ humid atmosphere. All culture reagents were purchased from Invitrogen (Cergy Pontoise, France). Green fluorescent protein (GFP)-expressing cells were obtained by transduction of cells with lentiviruses encoding for GFP.

### Mice Models

All animal use procedures were in strict accordance with the recommendations of the European Community (86/609/CEE) and the French National Committee (décret 87/848) for the care and use of laboratory animals. All used mice were female nude mice weighing approximately 23 g and housed under standard conditions with food and water *ad libitum*. To minimize the autofluorescence background signals, the mice were nourished with chlorophyll-free diet (Diet 210, SAFE, France) for 2 weeks before imaging. For the tumor xenograft model, a syringe was prepared containing 10^6^ tumor cells MDA-MB231 in a volume of 100 μl of phosphate-buffered saline (PBS) with 100 μl of Matrigel (BD Bioscience, Le Pont de Claix, France) at 0 °C. Cells were subcutaneously implanted between the omoplates of anesthetized mice and allowed to grow for several weeks until the desired tumor size was reached. For injection of probes and during imaging experiments, mice were anesthetized with isoflurane—1.25 % in a 1:3 mixture of O_2_ and air.

### PET/fDOT/CT Multimodal Imaging

For all experiments, ~7.4 MBq (200 μCi) of [^18^F]FDG were intravenously (IV) injected in anesthetized mice prior to imaging. For the first experiment, 2 nmol of the IRDye800CW 2-DG (Licor, Lincoln, Nebraska, USA) probe were IV administrated 24 h prior to [^18^F]FDG. The same protocol was used for the imaging of cathepsin activity using the probe ProSense680 (Perkin Elmer, Waltham, Massachusetts, USA). For the imaging of αvβ3 integrin, 5 nmol of the Arg-Gly-Asp (RGD)-based fluorescent probe Angiostamp (Fluoptics, Grenoble, France) was IV administrated 4 h before imaging.

The imaging procedure started with a PET scan 60 min after the injection, in accordance with the stabilization of the [^18^F]FDG inside the tumors. The mouse was, thereafter, transported and positioned in the fDOT instrument by means of a custom-made mouse support, all without moving the mouse in regard to the support. A local fDOT scanning was performed in the tumor area. Finally, for the case of ProSense680 and Angiostamp, the mouse was placed into a CT instrument for X-ray structural imaging.

PET acquisitions were performed using a MicroPET Focus 220 scanner (Siemens-Concorde Microsystems). The PET reconstructions were performed using the MicroPET Manager software (Siemens-Concorde Microsystems), based on a filtered back-projection algorithm. Images were reconstructed with the following frame durations: 2 × 15 min, suitable for the monitoring of [^18^F]FDG. The dimensions of reconstruction volumes were 256 × 256 × 95 × (number of time frames) with a voxel size of 0.475 × 0.475 × 0.796 mm^3^. For the fDOT measurements, a free-space fluorescent tomographic system, operating in the transillumination mode (Cyberstar, France), was used [18]. This imager incorporates a continuous wave laser mounted on a two-dimensional motorized stage for the optical scanning of the targeted region. A sensitive charge-coupled device (CCD) camera (C4742, Hamamatsu, Japan) with an objective lens focusing on the other side of the subject acquires images at the different laser positions. The system incorporates a band pass emission filter 720 ± 15 nm (HQ720/30, Chroma Technology, USA) for the detection of the ProSense680 and Angiostamp fluorophores and a 770-nm-long pass filter (770ALP Emitter XF3115, Omega Filters, USA) for the detection of IRDye800CW 2-DG. Since the fDOT scanner is mainly suited for Cy5.5-like fluorophores, we chose to test its sensitivity for imaging the 800-nm IRDye800CW 2-DG. In experiments where capillaries of controlled concentrations of the probe were imaged after being subcutaneously positioned in mice, the concentration limit at this wavelength was found in the order of 0.6 μM which is much lower than typical IRDye800CW 2-DG signals in tumors (in the range between 2 and 8 μM).

The tumor scan consisted of a square grid of 6 × 6 sources in steps of 2 mm. The geometry of the mouse was reconstructed with the use of a laser pattern method [16]. A 2 × 2 binning is applied on the camera pixels, and typical detector areas are in the order of 13 × 13 mm^2^ covering the targeted tissue area. As soon as the collection of projections was completed, the data were mathematically processed to give a 3D image of the fluorescence activity inside the mouse body (voxel size of 0.67 × 0.67 × 1 mm^3^) according to the procedure described in detail by Hervé *et al*. [18]. The resolution of the reconstructed signal is in the order of 1 mm^3^ [16].

The CT measurements were performed using the SkyScan 1178 high-throughput microCT (Skyscan, Kontich, Belgium). A total of 360 images were recorded with an angular step of 1°. The reconstructions were performed with the NRecon software (Skyscan, Kontich, Belgium). For each reconstruction, a total of 509 coronal slices were reconstructed with 0.1609-mm spatial interval between adjacent slices.

### Coregistration Toolbox

The mouse-supporting system contained four multimodal sources of contrast (model MMS10-068-1U; Isotope Product Laboratories, Valencia, CA, USA), which can serve as fiducial points for the coregistration of independently acquired images. For the coregistration of images between PET, fDOT, and CT, an in-house toolbox was built under the platform of the BrainVISA imaging software (http://brainvisa.info/index_f.html). The PET image from each mouse has been chosen as reference, and corresponding CT and optical images have been spatially aligned to it. First, CT and optical images have been flipped in order to fit with the orientation of microPET image. Once the fiducial markers are identified, their position is manually outlined. Images from each modality are then automatically coregistered with respect to the four fiducial marker positions by a least squares regression. From this regression, the optimal rotation and translation parameters can be calculated and stored as rigid body transformation matrix [16]. The errors of this coregistration procedure are below the resolution limit of PET and fDOT [19]. The fusion of images was also performed by using the BrainVISA software with the aid of the coregistration transformation matrices. Volumes of interest (VOIs) were drawn manually and were applied to each modality by the transformation matrices calculated from the process of image coregistration.

### *Ex Vivo* Analysis

At the end of the *in vivo* imaging protocol, mice were euthanatized by an overdose injection of sodium pentobarbital. For immunohistochemistry, the xenografts were removed surgically; incubated in zinc fixative (BD Pharmingen, #552658) at 4 °C during 24 h and then in 20 % sucrose with 4 % sodium phosphate-buffered paraformaldehyde (Labonord, #11699408; pH 7.4, 0.1 M) at 4 °C during 24 h, before being frozen in isopentane; and stored at −80 °C. Sections of 5-μm thickness were prepared on a cryostat, fixed in 4 % sodium phosphate-buffered paraformaldehyde, and permeablized in methanol–acetone (1:1, −20 °C, 5 min) and in phosphate-buffered saline 0.1 % triton × 100 (room temperature (RT), 5 min). After saturation of nonspecific binding sites with solution containing 5 % bovine serum albumin and 0.5 % Tween 20, tissues were incubated with primary antibody rabbit antihuman/rat GLUT-1 (Thermo Scientific, #RB-9052-P0, 1/200, RT, 1 h) diluted in saturation solution. Following PBS washes (three times), sections were incubated with AF546-labeled goat anti-rabbit IgG (H + L) (Invitrogen, A11010, 1/1000, RT, 30 min) diluted in saturation solution. Following three additional washes, sections were mounted with a Prolong Antifade kit (Molecular Probes, Invitrogen, P36930). Multichannel panoramic images were acquired at × 100 magnification on an epifluorescence-inverted microscope Axio Observer Zeiss (Zeiss, Germany) equipped with motorized stage.

For whole animal axial imaging, the euthanized mice were frozen at −80 °C for several hours. Then, whole-body axial sections at the level of the tumor of 150-μm thickness have been cut with a Leica CM 3050 cryostat (Leica Microsystems, Wetzlar, Germany). Whole-body sections subject to deforming, and therefore, care has been taken to select the least deformed sections. Five equally spaced axial sections, covering the whole tumor lump, were chosen and placed on microscope glass slides. The sections were placed in a planar imaging system (Photon Imager, Biospace, France) for the GFP imaging, in the planar imaging mode of the fDOT imager for the whole animal section imaging of ProSense680 and Angiostamp and in the planar fluorescence imager FluoBeam800 (Fluoptics, Grenoble, France) for the imaging of IRDye800CW 2-DG. Coregistration between the independently taken images has been performed by using the four edges of the glass slides as fiducial markers.

## Results

### Fusion of [^18^F]FDG PET Imaging with fDOT Imaging of Fluorescently Labeled IRDye800CW 2-DG

In the past decade, several fluorescent derivatives of 2-deoxy-d-glucose (2-DG) have been developed to provide a mimicking of [^18^F]FDG that can be used for fluorescence imaging of tumors [20]. One of these derivatives, IRDye800CW 2-DG, a 2-DG labeled with a NIR dye, demonstrated, using planar fluorescence imaging, high *in vivo* uptake by various tumors implanted subcutaneously in immunodeficient mice [21]. In these studies, it was observed that the fluorescent tracer provides a better contrast 24 h postinjection, while in the case of [^18^F]FDG, high contrast are generally achieved only 30 min to 2 h postinjection.

**Fig. 1:**
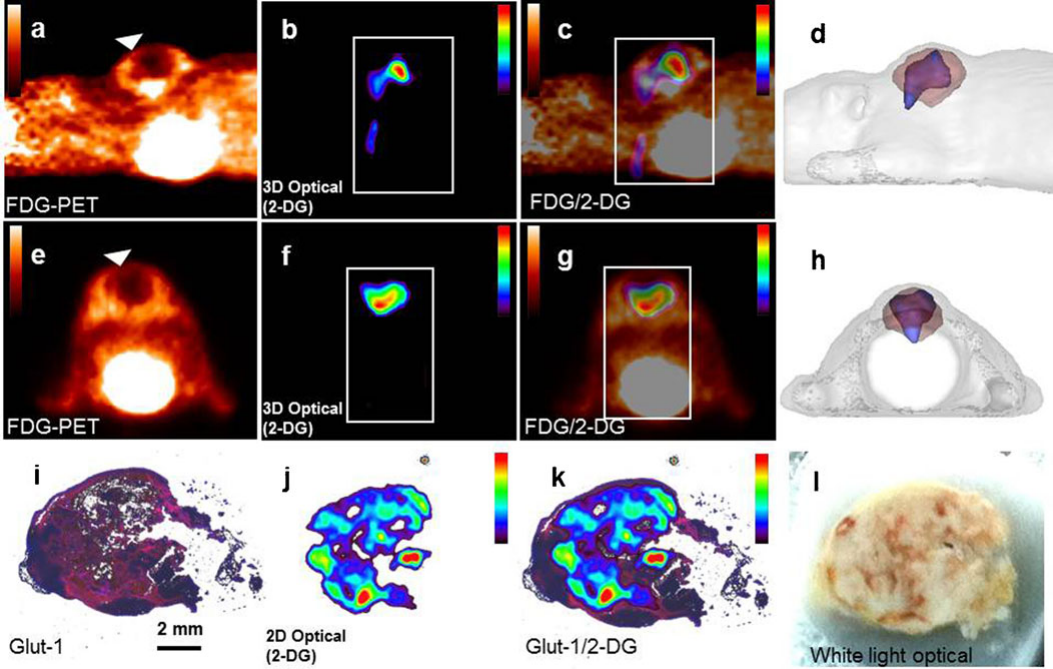
Imaging using IRDye800CW 2-DG and [^18^F]FDG. (**a**) Sagittal view of the [^18^F]FDG-PET signal of a xenografted mouse showing high activity at the site of the xenografted tumor (tumor volume of 775 mm^3^). The *arrow* pinpoints the position of the tumor as identified by [^18^F]FDG. (**b**) The fDOT IRDye800CW 2-DG signal at the corresponding sagittal plane. The area, in which the fDOT reconstruction is performed, is depicted by a *dotted white* *frame*. (**c**) Fused PET/fDOT sagittal image originating from the images of (**a**) and (**b**). (**d**) Sagittal view of the volumes occupied by the fDOT (*blue*) and PET (*red*) signal rendered to the 3D surface of the mouse (*gray*). (**e**, **f**, and **g**) The equivalents of (**a**), (**b**), and (**c**) are presented, but in the axial view; (**h**) Axial view of fused fDOT/CT image at the level of the tumor. (**i**) Immunohistochemical labeling for GLUT-1 expression (*in red*) in the excised tumor. Nuclei (*in blue*) are counterstained with DAPI. (**j**) Fluorescence image shows the biodistribution of IRDye800CW 2-DG signal in the same section than (**i**). (**k**) Image of the fusion of (**i**) and (**j**) shows the co-localization of the GLUT-1 and the IRDye800CW 2-DG. (**l**) White light image of the whole animal axial section.

To study the difference and similarities between these both tracers with 3D imaging, we coregistered images obtained by fDOT using IRDye800CW 2-DG with [^18^F]FDG PET images. As a model, we used subcutaneous tumor xenografts of human breast cancer cells (MDA-MB-231) subcutaneously implanted in nude mice. IRDye800CW 2-DG was first intravenously injected in mice (*n* = 6) bearing tumors of a size around 519 ± 223 mm^3^ (diameter 9.6 ± 1.2 mm). After 24 h, mice were IV injected with [^18^F]FDG just before a sequential fDOT/PET imaging acquisition. Results showed that each tracer demonstrated a higher uptake in tumors compared to surrounding tissue, leading to a clearly detectable imaging contrast for both PET and fDOT imaging (Fig. 1a, b, e, and f). However, the fused fDOT/PET images showed that IRDye800CW 2-DG and [^18^F]FDG displayed a significant different localization in the tumors (Fig. 1c, d, g, and h). [^18^F]FDG had a higher accumulation at the periphery of the tumor, while it had a lower uptake in the center of the xenografts.

Nevertheless, it has been proven *in vitro* that the uptake of IRDye800CW 2-DG by cancer cells can be specifically blocked by an antibody against GLUT1 glucose transporter or by excess of unlabeled 2-DG or d-glucose [21]. Hence, we decided to compare the localization of IRDye800CW 2-DG with the expression of GLUT1 by immunohistochemistry. Very low expression of GLUT1 was detected at the border of the tumor, while GLUT1 seems to be heterogeneously expressed in the center of the tumor. Interestingly, a good colocalization was also observed between GLUT1 and the IRDye800CW 2-DG (Fig. 1i–k).

### Fusion of [^18^F]FDG PET Imaging with fDOT Imaging of Cathepsin Activity

In another set of experiments, [^18^F]FDG PET imaging was combined with fDOT imaging of protease activity. Extracellular proteases are produced either by cancer cells themselves or by neighboring host cells. They participate to cellular invasion of basement membranes and connective tissue stroma as well as in the formation of new blood vessels during angiogenesis to support the burgeoning energy demands of rapidly growing tumors [22]. Hence, it can be interesting to study in parallel glucose metabolism and protease activity of tumors. For this purpose, we use an activatable optical probe (ProSense680, excitation maximum at 680 nm and emission maximum at 700 nm), which has the property of being optically silent (quenched) in its native state and can be specifically degraded by the cathepsins B, L, and S and plasmin, thereby generating an NIR fluorescence signal [23]. In that case, the fluorescence signal is not only indicative of the presence of this class of proteases but also of their activity.

**Fig. 2:**
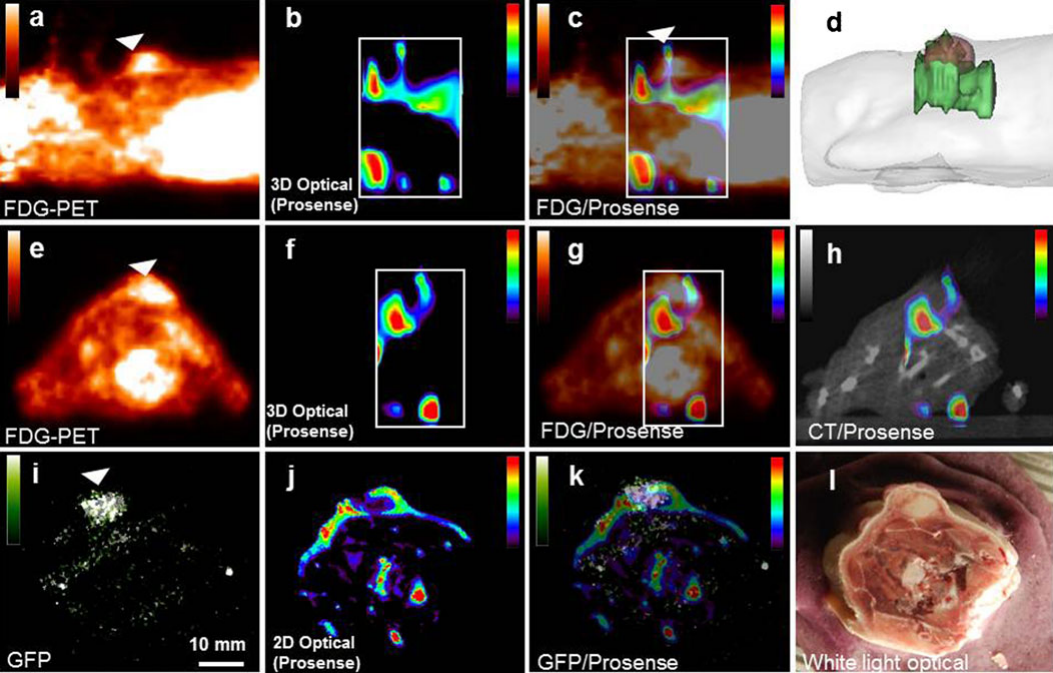
Imaging using ProSense680 and [^18^F]FDG. (**a**) Sagittal view of the [^18^F]FDG-PET signal of a xenografted mouse showing high activity at the site of the xenografted tumor (tumor volume of 50 mm^3^). The *arrow* pinpoints the position of the tumor as identified by [^18^F]FDG. (**b**) Sagittal fDOT image of ProSense680 activity. The area in which the fDOT reconstruction was performed is depicted by a *dotted white* *frame*. (**c**) Fused PET/fDOT image highlighting the distribution of the optical probe with respect to the PET nuclear signal. (**d**) Reconstructed mesh volumes of fDOT signal (*green*) and PET signal (*red*) rendered to the envelope of the mouse (gray); (**e**, **f**, and **g**) are the equivalent of (**a**), (**b**), and (**c**), but in the axial view. (**h**) Axial view of fused fDOT/CT image at the level of the tumor. (**i**, **j**, **k**, and **l**) Planar images of axial animal sections. (**i**) GFP imaging enabling the visualization of the GFP tumor cells. (**j**) Prosense680 imaging of the same section. (**k**) Fusion image of (**j**) and (**k**), showing that the cathepsin activity, is predominantly located outside the tumor in accordance to the *in vivo* case. (**l**) White light image of the section.

Nude mice, bearing subcutaneous tumor xenografts of human MDA-MB-231 cells stably transformed to express green fluorescent protein, were firstly injected intravenously with ProSense680. Then, after 24 h, a second IV injection of [^18^F]FDG was performed followed by a sequential fDOT/PET/CT acquisition. From the fused fDOT/PET/CT images (*n* = 3), we observed that, in contrary to the IRDye800-2DG, the cathepsin activity highlighted by ProSense680 was predominantly located underneath the tumor highlighted by FDG signal (Fig. 2a–h). To confirm this data, whole-body axial sections (~150 μm) of euthanized mice were performed with a cryostat at the level of the tumor and imaged by planar fluorescence imaging. In this case, the localization of protease activity was compared with the localization of tumor cells detected by their GFP signal. The distribution of ProSense680 signal clearly localized at the margins of the tumor tissue (Fig. 2i–k). Hence, *ex vivo* imaging confirmed *in vivo* imaging, although PET and fDOT imaging have a lower spatial resolution (~0.8 mm), which explains why NIRF fluorescent signal surrounding the tumor (Fig. 2g) appears thicker in the *in vivo* fDOT images compared to the *ex vivo* section analysis (Fig. 2g and k, respectively). These results suggest that the protease activity is higher in the stromal tissue surrounding the tumor than in the tumor region rich in cancer cells.

**Fig. 3:**
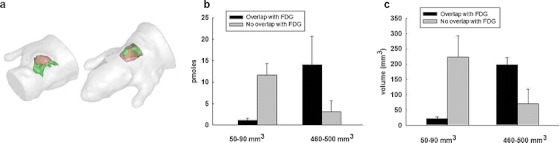
Distribution of the ProSense680 signal into tumors of different sizes. (**a**) Representative mesh volumes of ProSense680 (*green*) and [^18^F]FDG (*red*) for the case of a tumor with a volume of 50 mm^3^ (*left*) and 450 mm^3^ (*right*). (**b** and **c**) Histograms evaluating the overlapping between the fluorescent signal and the [^18^F]FDG for quantity (**b**) and volume (**c**) for the cases of small and big tumors (*n* = 3).

The above results were obtained for the case of small tumor having a volume measured by [^18^F]FDG PET imaging around 63 ± 24 mm^3^ (corresponding to tumor diameters of 4.8 ± 0.6 mm). In order to study the evolution of protease activity upon tumor growth, we performed the same fDOT/PET imaging experiments on mice bearing more developed tumor xenografts with an average size around 483 ± 21 mm^3^ (*n* = 3). Although the tumor volume highlighted by [^18^F]FDG was seven times larger, the same level of protease activity was measured both in terms of the volume and the quantity of fluorescence signal (246 ± 48 mm^3^ with 15.0 ± 0.6 pmol and 269 ± 52 mm^3^ with 15.9 ± 0.3 pmol for the small and the big tumors, respectively). In contrast, the distribution of the fluorescence signal with respect to the [^18^F]FDG was completely changed (Fig. 3). In that case, the fluorescence was mostly located inside the volume covered by the [^18^F]FDG signal, while it was previously found beneath the tumor. Up to 75 % of the fluorescence signal was colocalized with the [^18^F]FDG signal in comparison to ~7 % for small tumors. In conclusion, fDOT/PET imaging suggests that the distribution of the protease activity could vary during the tumor growth in this tumor model, although its intensity is not increased.

### Fusion of [^18^F]FDG PET Imaging with fDOT Imaging of Fluorescent RGD-Based Probe

Several ligands (like peptides, antibodies, or aptamers) are developed to target specific membrane proteins overexpressed in tumors. These ligands can be useful tools not only to build contrast agents for cancer imaging but also to specifically address drugs. fDOT imaging is still increasingly used to validate these ligands *in vivo* [17, 24, 25]. However, we hypothesize that the fusion of fDOT with PET imaging can further improve this validation enabling a direct comparison with PET tracers that are already used in clinic. As a model, we decided to compare the distribution of the RGD-based fluorescent probe Angiostamp680 with [^18^F]FDG PET imaging. The RGD peptide is known to bind the αvβ3 integrin, a protein overexpressed at the surface of certain cancer cells as well as at the surface of endothelial cells during angiogenesis [26]. Angiostamp680 is composed of four cRGD cyclopeptides linked to a cyclodecapeptide platform labeled with a NIR fluorescent dye [27]. This tracer has previously demonstrated a rapid uptake in tumors after IV injection and represents a promising contrast agent for fluorescence imaging-assisted surgery [28].

**Fig. 4:**
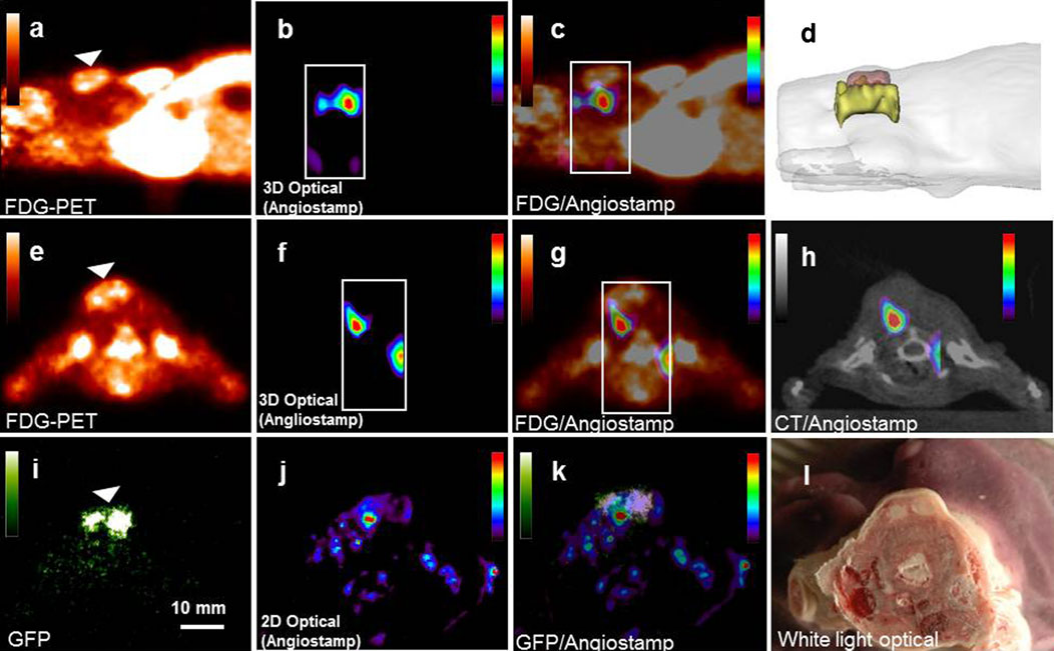
Imaging using Angiostamp and [^18^F]FDG. (**a**) Sagittal slice of [^18^F]FDG PET at the level of the tumor (with a tumor volume of 59 mm^3^). The *arrow* pinpoints the position of the tumor as identified by [^18^F]FDG. (**b**) Sagittal fDOT image of Angiostamp. The area, in which the fDOT reconstruction is performed, is depicted by a *dotted white* *frame*. (**c**) Fused PET/fDOT image highlighting the distribution of the optical probe with respect to the PET nuclear signal. (**d**) Reconstructed mesh volumes of fDOT signal (*green*) and PET signal (*red*) rendered to the envelope of the mouse. (**e**, **f**, **g**, and **h**) The equivalent of (**a**), (**b**), (**c**), and (**d**), but in the axial view. (**i**, **j**, **k**, and **l**) Planar images of axial animal sections. (**i**) GFP imaging enabling the visualization of the GFP tumor cells. (**j**) Angiostamp imaging of the same section. (**k**) Fusion image of (**j**) and (**k**) showing that the protein activity is predominantly located outside the tumor in accordance to the *in vivo* case. (**l**) White light image of the section.

**Fig. 5:**
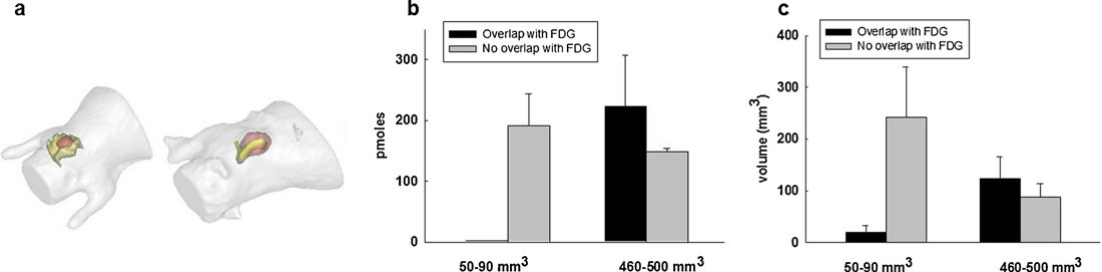
Distribution of the Angiostamp signal into tumors of different size. (**a**) Representative mesh volumes of Angiostamp (*yellow*) and [^18^F]FDG (*red*) for the case of a tumor with a volume of 40 mm^3^ (*left*) and 220 mm^3^ (*right*). (**b** and **c**) Histogram evaluating the overlapping between the fluorescent signal and the [^18^F]FDG for quantity (**b**) and volume (**c**) for the cases of small and big tumors (*n* = 3).

Angiostamp680 was IV injected in nude mice bearing subcutaneous tumor xenografts of human MDA-MB-231 cells expressing GFP (*n* = 3). After 3 h, [^18^F]FDG was IV injected just before a sequential fDOT/PET imaging acquisition. As previously described, we compared the results obtained in nude mice bearing either small or big tumors (~44 ± 16 and ~230 ± 20 mm^3^ calculated from [^18^F]FDG imaging, respectively). Similarly to ProSense680, we observed that the Angiostamp680 lies below the signal of [^18^F]FDG for small tumors (Fig. 4a–h). The localization of the probe mostly outside the tumor of small size was also confirmed *ex vivo* by planar fluorescence imaging of whole-body axial sections (Fig. 4i–k). Contrary to the case of small tumors, for big tumors, Angiostamp is much more localized inside the volume covered by the [^18^F]FDG signal (~57.9 ± 10.9 % of the fluorescence signal is colocalized with [^18^F]FDG in contrary to only ~6.7 ± 5.0 % for smaller tumor sizes, see Fig. 5). However, compared to ProSense680, there is an increase of the total amount of the Angiostamp680 in the biggest tumors (450 ± 148 pmol compared to 206 ± 67 pmol, for the big and the small tumors, respectively, Fig. 5), although it was restricted in a similar or slightly smaller volume (211 ± 58 mm^3^ compared to 261 ± 95 mm^3^). These results suggest that, for this model, the αvβ3 integrin is predominantly present in stromal tissue of small tumors, and its expression is increased and much more localized inside the tumor during its growth.

## Discussion

Recently, the fusion of small animal PET imaging with 3D fluorescence diffuse optical tomography has been considered as a promising approach [29, 30]. This method has been previously used to calibrate fDOT using PET [16, 17]. Here, we demonstrated that it could also be used for cross-validation of imaging methods in both modalities or to provide synergic information monitoring several molecular pathways inside the same subject. As a proof of principle, we studied the additional benefits provided by the combination of fDOT with PET imaging by comparing the biodistribution of [^18^F]FDG with three fluorescent probes in nude mice bearing tumor xenografts of MDA-MB-231 cells. [^18^F]FDG is the most used PET tracer in the world [1]. It provides information on glucose metabolism, which is particularly useful for the detection of tumors as well as to evaluate the effect of drugs. Since [^18^F]FDG is so valuable in oncology, there is a high interest for developing fluorescent analogs of this tracer, which can be used for small animals studies using optical methods.

Our first experiment concerned the use of PET/fDOT imaging to evaluate a fluorescent 2-deoxy-glucose, the IRDye800CW 2-DG. In our model, IRDye800CW 2-DG was mostly localized at the center of the tumor, whereas [^18^F]FDG was much more accumulated at the periphery of the tumor. These results suggest that IRDye800CW 2-DG may not be a surrogate probe for [^18^F]FDG. Accordingly, Tseng *et al*. recently demonstrated in another tumor model that the two probes show a quite different imaging of treatment response [31]. Nevertheless, it should be noticed that, as in our study, both probes were not compared at the same time after injection (24 h and few minutes after injection for IRDye800CW 2-DG and [^18^F]FDG, respectively). Hence, further studies are warranted to compare this fluorescent tracer with [^18^F]FDG at the same time. However, our results clearly demonstrates the benefit of fDOT/PET imaging for cross-validating or invalidating probes that are developed to be used for the same applications in both modalities. This approach could be particularly useful to validate new fluorescent probes that are developed for clinical application in the field of endoscopy and imaging-guided surgery [32], comparing their distribution with PET tracer already being used in patients.

Furthermore, fDOT/PET imaging can also provide additional benefits using different probes in both modalities to monitor in parallel different processes *in vivo* in the same subjects. This approach can be especially useful to complement PET imaging with fluorescent probes and imaging approaches that were reserved to fluorescence microscopy until now. For instance, it is well known that fluorescence of dye can be modulated by their environment (pH, hydrophobicity, *etc*.) or their interaction with nearby molecules, leading to fluorescence quenching or resonance energy transfer. This property has been widely used to develop so-called smart or activatable probes whose fluorescence can be switched “on” or “off” depending on a molecular process, such as interaction with a biomarker or degradation by a specific enzyme [23]. Such activatable probes cannot be developed with radiotracers that are always on and whose signal intensity is only dependent of isotope decay. Here, we combined [^18^F]FDG imaging with a fluorescent activatable probe that allowed us to monitor the activity of cathepsins. The activity of proteases is highly regulated at posttranslational stages and requires specific localization in cells as well as proteolytic maturation. Hence, the use of fluorescent activatable probes represents a promising approach to study their enzymatic activity directly *in vivo* compared to classical tracers that were developed to study their expression. In our tumor model, there has been some evidence that the activity of cathepsins does not increase during tumor growth, but its localization was changed. Therefore, fusion with [^18^F]FDG imaging allowed us to observe that the fluorescence was first predominantly located beneath the tumor before being mostly localized inside the tumor during its growth. Such information would be impossible to obtain with fDOT alone and could be extended with many other fluorescent activatable probes that have been developed to monitor the activity of different classes of proteases. Nevertheless, it is obvious that these data will have to be complemented by other experiments to verify that the signal does not correspond to a difference in the diffusion of the fluorescent probe inside the tumor. One possibility can be provided once again by PET/fDOT imaging using fluorescent activatable probes radiolabeled with positrons emitters. In that case, it might be possible to normalize the fluorescence signal corresponding to the enzymatic activity with its distribution measured by PET. Ongoing efforts are currently being conducted to develop such multilabeled probes, also named monomolecular multimodality imaging agents [33].

Finally, other benefits of PET/fDOT imaging might be to study the effect of drugs in small animal models. For instance, we were able to complement [^18^F]FDG PET with fDOT imaging of a fluorescent ligand that binds αvβ3 integrin. This protein is a known marker of angiogenesis. In our model, results indicated an increase of fluorescence signal during tumor growth and a localization that was first predominantly below the tumor before expanding toward the tumor during its growth. This result was expected since the growth of tumors requires the formation of new blood vessels from preexisting vessels, which are mainly present in the stroma at the beginning of tumor growth. Therefore, such PET/fDOT imaging might be useful to study the effect of antiangiogenic therapies, measuring in parallel the effect on an angiogenic marker and a metabolism marker.

In the present study, we used a simple approach by moving a mouse support between different instruments. However, other groups are attempting to build an integrated apparatus that can perform at the same time both optical and nuclear imaging and at the same time using rings of separate scintigraphy detectors and CCD cameras [34], combined PET or gamma camera with an optical detector [35, 36] or adaptation of a conical mirror-based fDOT inside the PET gantry [37, 38]. Such instrument could improve fDOT/PET imaging and will be useful to perform dynamic imaging.

## Conclusions

Here, we demonstrate the additional benefits of combined fDOT and PET imaging, comparing the biodistribution of FDG with three fluorescent probes in xenograft tumor models. This technique was used to show the advantages of the combined technique for the evaluation of new optical probes developed as surrogate markers to PET probes. We also showed that fusion of PET with fDOT imaging could provide unique benefits to monitor in parallel different physiological processes at the same time.
